# Primary Cutaneous Hodgkin's Lymphoma: An Extremely Rare Entity

**DOI:** 10.7759/cureus.19272

**Published:** 2021-11-05

**Authors:** Darine Hakkou, Ismail Belefqih, Samia Sabri, Siham Dikhaye, Houda Bachir, Siham Hamaz, Habiba Alaoui, Khalid Serraj

**Affiliations:** 1 Internal Medicine, Mohammed First University, Oujda, MAR; 2 Internal Medicine, Mohammed VI University Hospital, Mohammed First University, Oujda, MAR; 3 Dermatology, Mohammed VI University Hospital, Mohammed First University, Oujda, MAR

**Keywords:** chemotherapy, immunostaining, biopsy, hodgkin lymphoma, skin

## Abstract

Skin involvement in Hodgkin's lymphoma (HL) is rare. The diagnosis can be difficult, mainly due to the wide range of cutaneous lesions that can be observed, but also due to the differential diagnosis, even after the immunohistochemical staining. We present the case of a 30-year-old man who presented with a painful cutaneous nodular lesion; biopsy and immunohistochemical stains were consistent with classic HL. The patient was treated with adriamycin, bleomycin, vinblastin, and dacarbazine (ABVD) with complete remission.

## Introduction

Hodgkin's lymphoma (HL) is likely the rarest of all lymphomas involving the skin with a prevalence of 0.5% to 7.5% of all HL patients [1,2]. One of the most challenging questions in patients with skin HL is to distinguish between specific and non-specific lesions, which are part of the paraneoplastic manifestations [3]. We report the case of a patient that emphasizes the important role of skin biopsy both in the positive and differential diagnosis of cutaneous HL.

## Case presentation

**Figure 1:**
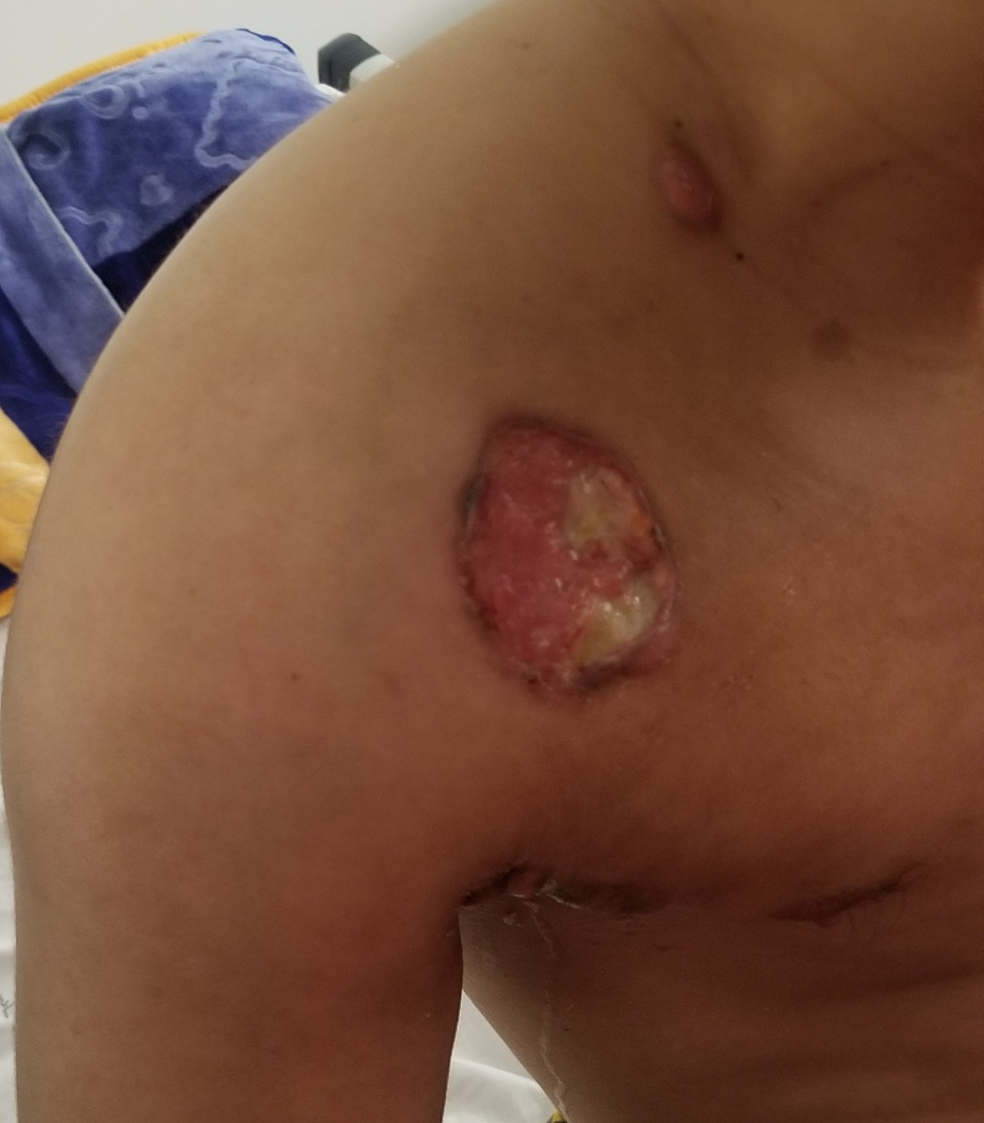
Pretreatment photograph showing the three skin lesions described above

**Figure 2:**
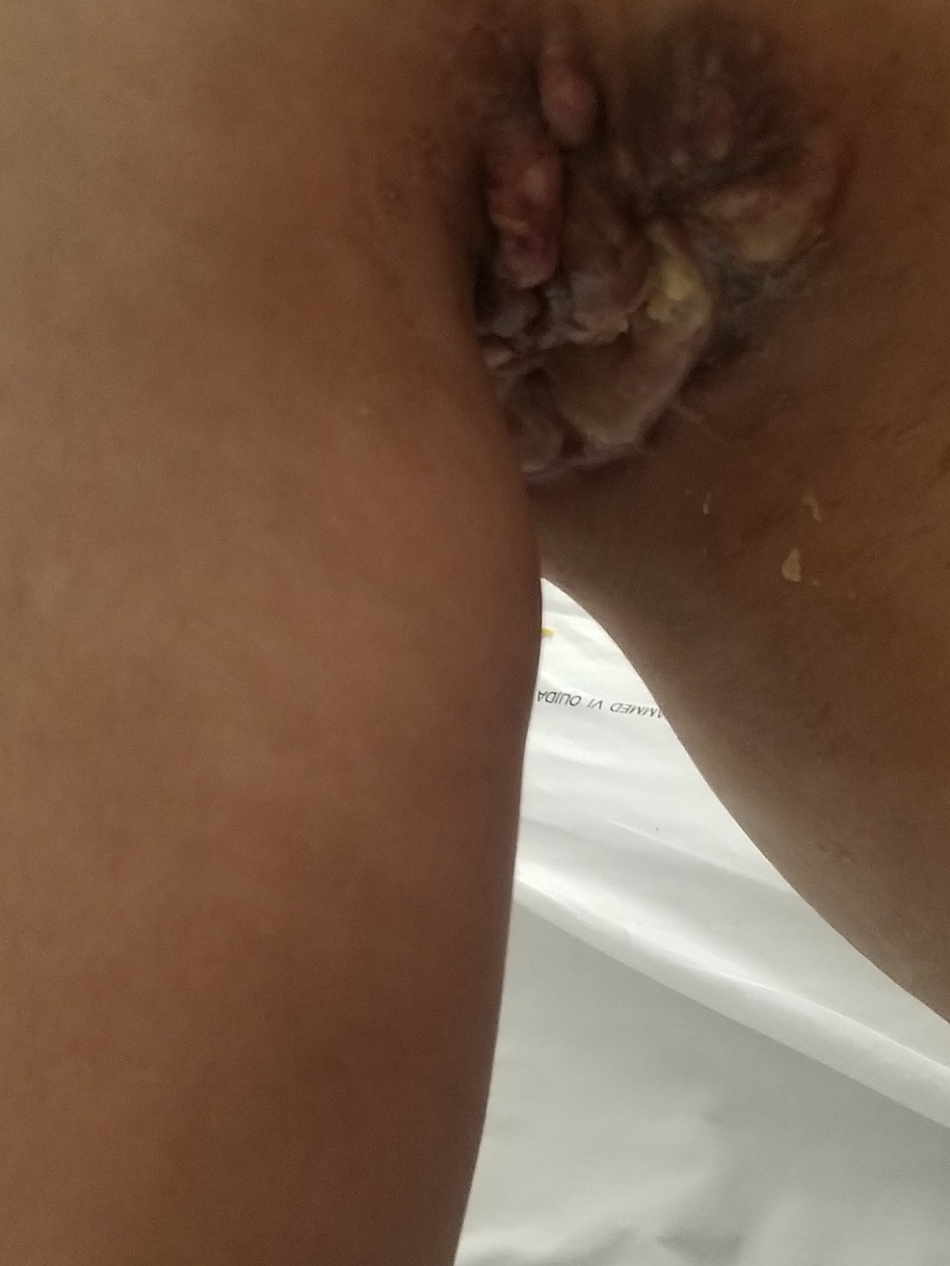
Image showing the lesion and the axillary mass

**Figure 3:**
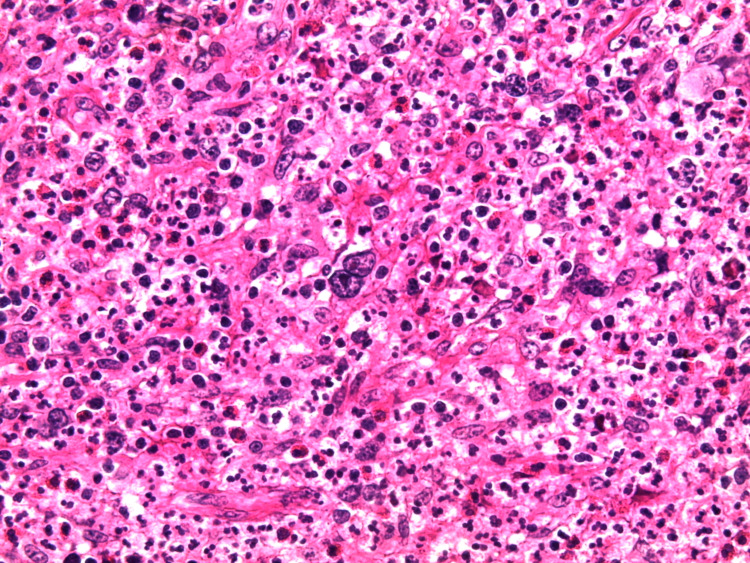
Skin biopsy with hematoxylin-eosin-safran (HES) coloration G x400 Polymorphic infiltrate with granulomatous background rich in small lymphocytes, histiocytes, plasma cells, neutrophils and eosinophils mixed with large lacunar Hodgkin skin cells + Reed Sternberg cells.

**Figure 4:**
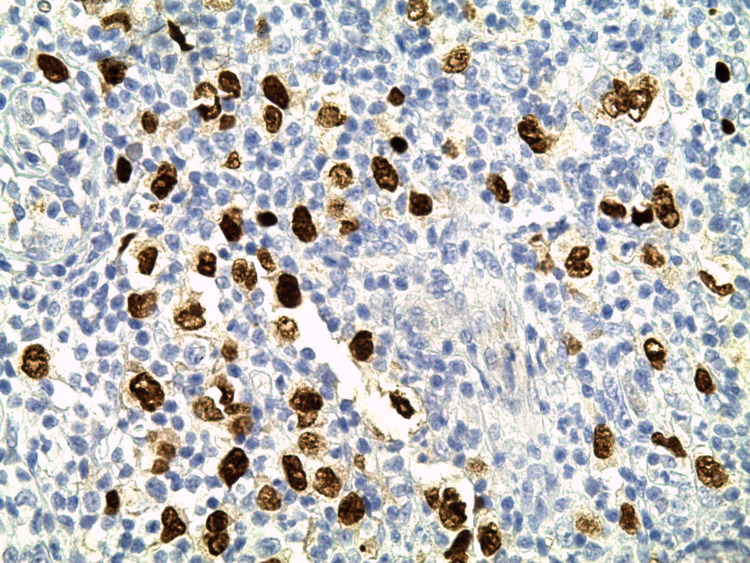
Image showing immunohistochemical study of the skin biopsy, G x400 CD30: membrane marking + golgitic densification of tumor cells.

**Figure 5:**
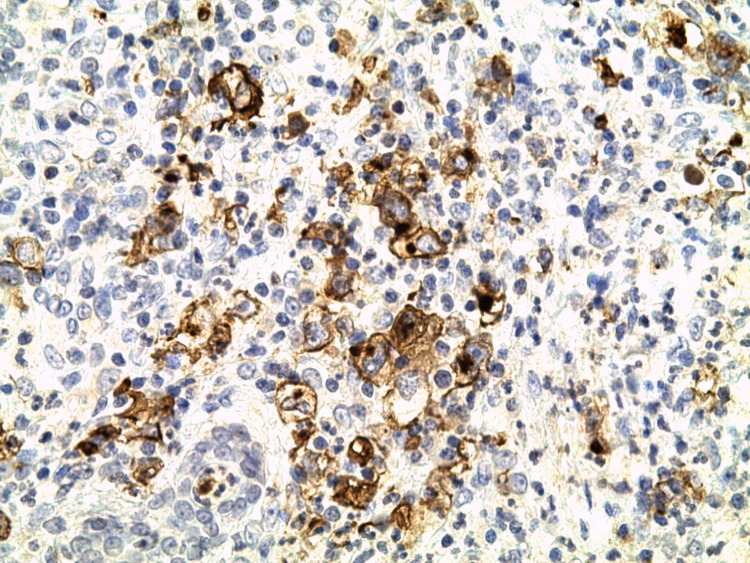
Image showing immunohistochemical study of the skin biopsy, G x400 Pax5 (+): nuclear expression.

**Figure 6:**
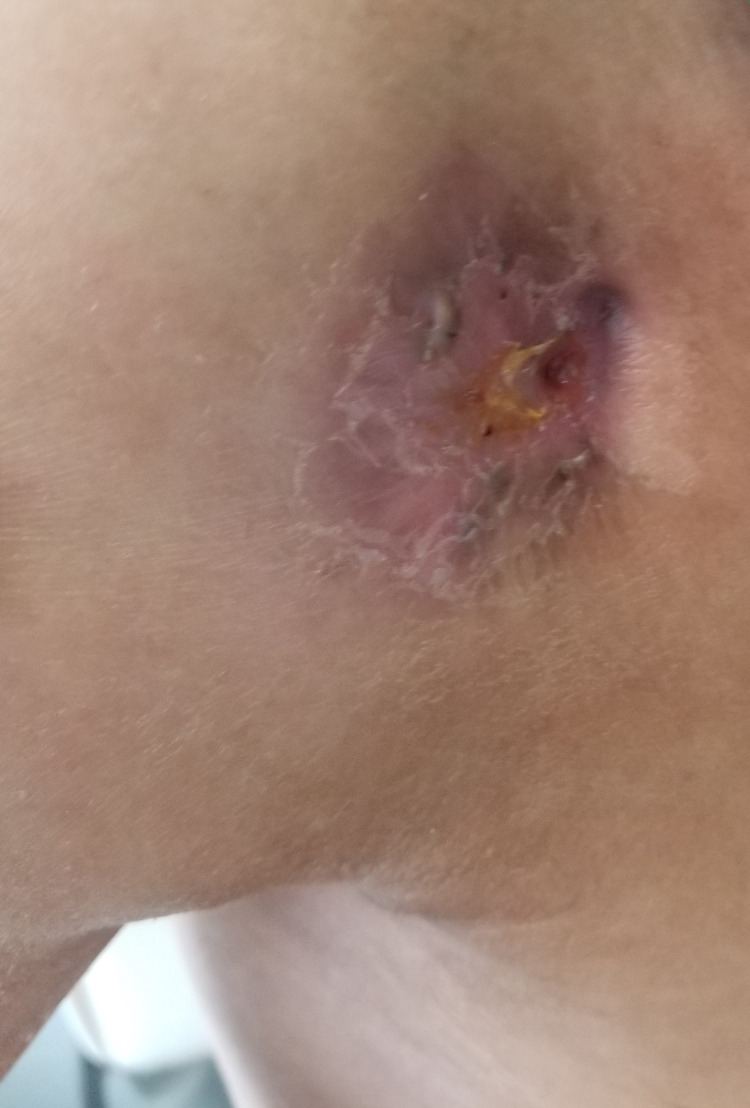
Image showing the evolution of skin lesions after two cycles of chemotherapy

**Figure 7:**
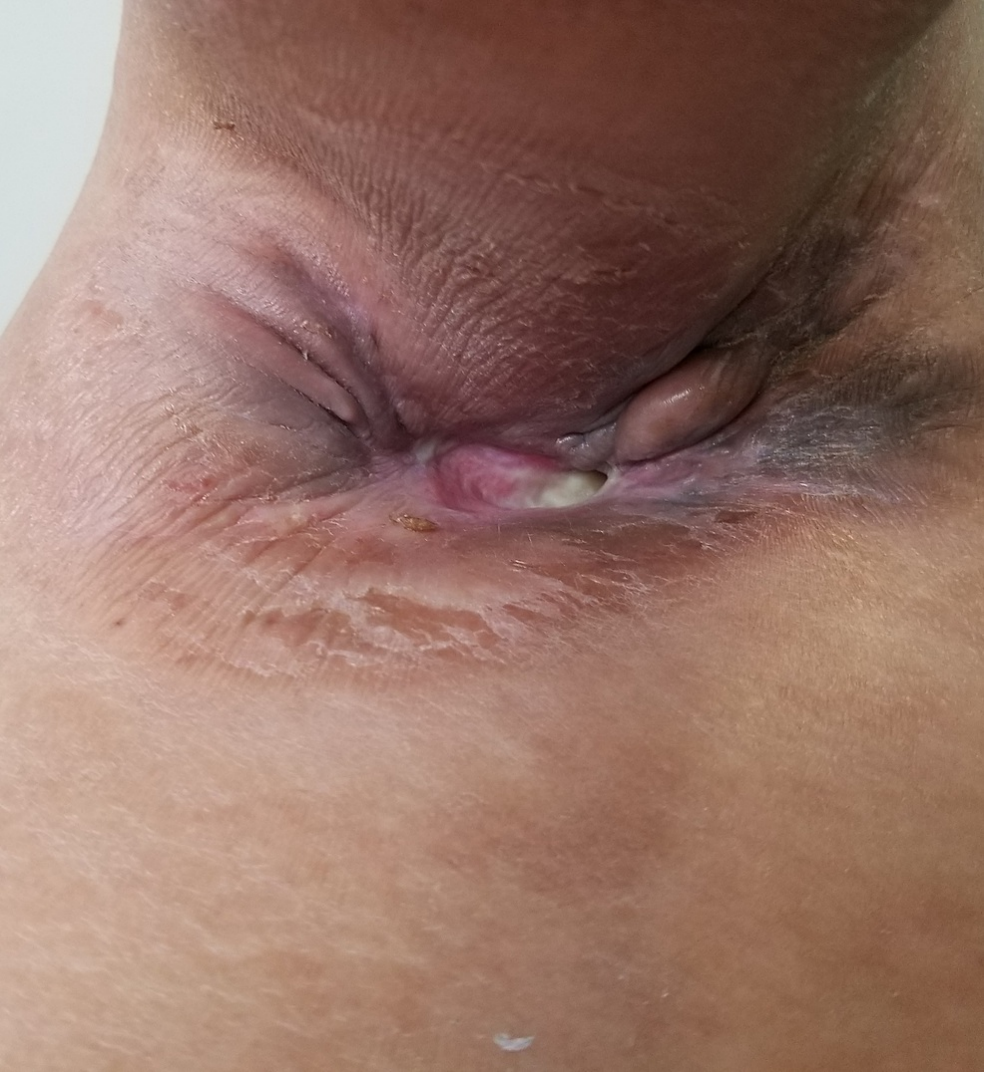
Image showing the evolution of the axillary mass after two cycles of chemotherapy

We report the case of a 30-year-old man, with a history of schizophrenia, who presented 16 months before the first admission; he had a painful nodular lesion progressively increasing in size at the right axillary cavity, initially neglected by the patient. The evolution was marked by fistulization, which warranted inpatient management. Clinical evaluation showed the presence of three skin lesions, as seen in Figure 1: an erythematous, non-ulcerated, non-fistulized nodular lesion measuring 20 x 8 mm at the right side of the base of the neck, a well-limited oval ulcerated lesion with a fibrinous and purulent background measuring 30 x 50 mm at the right subclavicular level, and an ulcerated lesion with a fibrinous and purulent background and a serosanguinous liquid discharge, ranging from 15 to 40 mm in the right axillary cavity. Skin lesions were accompanied by a right axillary mass, difficult to characterize because of the painful skin lesion homolateral (Figure 2). The rest of the lymph nodes were free. A skin biopsy initially showed nonspecific suppurative remodeling, thus the patient received antibiotic therapy without any improvement. A second skin biopsy was then performed and was in favor of HL with the presence of a proliferation made of large cells represented by Hodgkin's cells, sometimes of lacunar aspect, Reed Sternberg cells, and some mummified cells. These elements evolved within a granulomatous background rich in small lymphocytes and histiocytes with which are associated numerous plasma cells and neutrophilic leukocytes with eosinophilic polynuclear cells (Figure 3). The immunohistochemical study showed positive anti CD30, anti-CD15, anti-PAX 5, anti-Epstein-Barr virus (EBV)/latent membrane protein (LMP-1), negative anti CD20, and anti-CD3 (Figures 4-5). CT scan imaging had not shown other abnormalities, namely signs of tumoral syndrome, and the bone marrow biopsy excluded the involvement of bone marrow. Biological parameters and echocardiography did not find any abnormality, except for a biological inflammatory syndrome. The diagnosis of HL stage 1 according to Ann arbor classification was retained and the patient was treated with six cycles of ABVD protocol with rapid and good response (Figure 6-7).

## Discussion

HL represents 0.58% of all diagnosed cancers. There is a slight male predominance (1,1:1). The incidence of HL shows a bimodal peak with age between 25 and 30 years and between 75 and 80 years [4]. Non-specific skin involvement is seen in 17%-53% of cases [5]. The incidence of cutaneous-specific Hodgkin's disease has been estimated to range between 0.5% and 3.4%, with a reduction in incidence due to improved therapeutic means, and particularly the transplantation of stem cells [6]. The etiology and pathogenesis of this disease remain unknown. Some risk factors such as familial predisposition, EBV or human immunodeficiency virus (HIV) infection, and immune suppression have been implicated [7]. Three mechanisms have been proposed to explain the spread of systemic Hodgkin's disease to the skin: hematogenous dissemination, direct extension from involved lymph nodes, and retrograde lymphatogenous spread from involved proximal lymph nodes [6]. The most common means of transmission appears to be retrograde spread from the affected nodes, as most reported cases occur on areas of the skin drained by the affected lymph nodes, such as the chest and axilla, and the pathology generally does not demonstrate direct extension from an adjacent node [6]. It remains unclear whether primary HL is a distinct entity or whether it represents direct extension or hematogenous spread of the tumor [8].To establish a diagnosis of primary cutaneous HL, it must meet specific criteria, defined by the presence of HL in the skin with no lymph node involvement and no evidence of extracutaneous dissemination three months following the diagnosis [3]. Lesions of primary cutaneous HL have been described as erythematous papules and nodules with or without ulceration, most commonly involving the scalp and cervical region and extremities [9] (Table 1).

**Table 1:** Review of primary cutaneous Hodgkin's disease in the English literature [3]

Case	Age	Gender	Clinical presentation
1	54	M	Solitary nodule left lower leg.
2	52	M	Large, reddish-brown tumor nodule and papules on the right forearm.
3	17	M	Lifelong history of skin rash variously diagnosed as “dermatitis,” solitary nodular mass on right thigh.
4	50	M	3-month history of 2-cm cutaneous nodules symmetrically located on each lateral chest wall; 2 months later, 1.5 cm right inguinal lymph node recurrence.
5	45	F	3-month history of 1- to 2-cm nodules on forearms and legs.
6	86	M	Solitary left ankle mass.
7	34	F	Subcutaneous nodule left lower neck with draining sinus tract, mediastinal Lymphadenopathy developed 4 months later.
8	59	M	10-month history of painless, erythematous and indurated lesions on the skin of the left flank, inner thigh, and right dorsal foot.
9	70	M	6-month history of erythematous papules and nodules on the right back.
10	76	F	Multiple subcutaneous nodules on the head and neck.

The immunohistochemical diagnosis of primary cutaneous Hodgkin's disease is difficult, although the detection of neoplastic Reede-Sternberg cells with mirror nuclei, the positivity of CD30 and CD15 activation antigens, and the negativity of CD45 strongly support the diagnosis of Hodgkin's disease [10].There are many other diseases in which Reed-Sternberg cells can be found, including benign thymoma, proliferative myositis, infectious mononucleosis, lymphomatoid papulosis type A, anaplastic large cell lymphoma, and some forms of B-cell lymphoma, particularly cases expressing CD30 or associated with EBV infection and hematopoietic tumors [11-13]. In addition, the presence of CD15 is reported in a subset of lymphomatoid papulosis, mycosis fungoides, CD30+ large cell analplastic lymphoma and some T-cell lymphomas [14,15]. In contrast, PAX-5 expression is able to differentiate HL from other situations [3]. The first inconclusive biopsy in our patient emphasizes the importance of a good technical quality of the biospic gesture, of a deep biopsy with fragment consisting idelament an excision and the importance of immunostaining from the outset.The nonspecific skin manifestations of HL can present as a paraneoplastic syndrome, the most common symptoms are eczema, followed by pruritus/prurigo nodularis, mycosis fungoides, erythema nodosum, ichthyosis, areas of hyperpigmentation, and alopecia distinct from that often induced by chemotherapy [6,8]. In addition, there are unusual and nonspecific skin manifestations, such as lymphohistiocytic infiltrates, pyoderma gangrenosum, and atypical pityriasis rosae [8]. Furthermore, it is necessary to eliminate the specific infections mainly tuberculosis, especially in our endemic context and the presence of granuloma observed on the histological study in our patient. This is another reason to systematically perform immunostaining. We present a case that meets the diagnostic criteria to justify categorization as primary cutaneous HL. The histological presentation is consistent with the classic nodular sclerosing variant of the disease. The cutaneous manifestation encountered in this case is consistent with the cases reported previously (Table 1).

There is no standard therapy for cutaneous HL. It is usually decided on a case-by-case basis. Treatment options include systemic chemotherapy with or without radiotherapy, or local treatment with topical agents, or local radiotherapy alone. For cases reported in the literature of primary cutaneous HL, 20% of patients were treated with topical corticosteroids alone and showed excellent results. Local radiotherapy alone was administered in 20% of cases with a good local response followed by systemic progression after one to six years [16]. The remaining cases were treated with six cycles of systemic chemotherapy such as mechlorethamine, vincristine, procarbazine, prednisone (MOPP), ABVD. This resulted in a complete remission with a follow-up of more than three years, apart from a single case that progressed after two months [17,18]. In cutaneous HL associated with systemic involvement, the disease is classified as stage IV according to Ann Arbor staging and thus the standard treatment is chemotherapy with ABVD/bleomycin, etoposide, doxorubicin cyclophosphamide, vincristine, procarbazine, prednisolone (BEACOPP)/Stanford with or without involved field radiation [5]. Thereby, the therapeutic response is satisfied, but there is a need for further follow-up because of the high potential for recurrence. In our case, the therapeutic decision was a systemic chemotherapy type ABVD, given the late management of the skin involvement and the subsequent appearance of the right axillary mass. With a good evolution and tolerance of the treatment to date, but if the diagnosis was made in a timely manner, would a local radiotherapy be sufficient?

## Conclusions

Primary cutaneous HL is an extremely rare entity; whilst the association of HL with skin involvement is also rare, it is well described in the literature. Pathologic diagnosis using immunohistochemistry is necessary for diagnosis of isolated skin involvement. Despite the good prognosis of primary cutaneous HL described in the literature, long-term follow-up is essential to detect progression to systemic involvement.
